# Optimizing Sensor Number and Placement for Accurate and Robust Center of Pressure Estimation on Instrumented Insoles

**DOI:** 10.3390/s26092723

**Published:** 2026-04-28

**Authors:** Matthis Gautier, Fabien Parrain, Pierre-Yves Joubert

**Affiliations:** Centre de Nanosciences et de Nanotechnologies, Centre National de la Recherche Scientifique, Université Paris-Saclay, 91120 Palaiseau, France; fabien.parrain@universite-paris-saclay.fr

**Keywords:** gait analysis, smart insoles, center of pressure, sensor optimization, wearable sensors, plantar pressure, sparse sensing

## Abstract

Smart insoles equipped with pressure sensor matrices are increasingly used for gait analysis, yet high-density arrays compromise battery life and data throughput. This study aims to identify the optimal sparse sensor layout required to accurately estimate the Center of Pressure (CoP) by analyzing the trade-off between sensor number, spatial placement, and reconstruction error. Plantar pressure data were collected from twelve healthy participants walking at a self-selected speed using 16-sensor connected insoles. A combinatorial algorithm evaluated all $2^{16}-1$ possible sensor combinations to minimize the Root Mean Square Error (RMSE) in the antero-posterior, medio-lateral, and global Euclidean directions. Results reveal a non-linear convergence of accuracy that depends on the spatial axis. For longitudinal and global progression, a clear inflection point achieving sub-centimetric accuracy (RMSE < 5 mm) is reached at seven sensors. In contrast, medio-lateral tracking shows its largest discrete error reduction at five sensors, followed by gradual improvements at higher densities. Anatomical frequency analysis highlights distinct spatial requirements: the posterior heel is consistently selected for medio-lateral accuracy, while the lateral arch and metatarsal regions are critical for longitudinal progression. These findings suggest that while a minimum of seven strategically placed sensors enables robust CoP tracking across all spatial axes, optimal hardware design should remain task-specific. This work provides a data-driven framework for the development of energy-efficient wearable gait monitoring systems.

## 1. Introduction

Center of Pressure (CoP) analysis is a cornerstone in the assessment of human postural control and gait biomechanics, providing crucial insights for a variety of clinical applications, from the diagnosis of balance disorders to the design of prosthetics and orthotics [1,2]. Historically, the accurate measurement of CoP relied primarily on the use of force platforms, considered to be the gold standard in the laboratory [3,4,5,6]. However, the static and non-portable nature of these systems limited their application to controlled environments, preventing the assessment of CoP under natural, everyday motion conditions.

With the advent of miniature sensor technologies and embedded systems, instrumented insoles have become a portable and versatile alternative for measuring plantar pressure and estimating CoP coordinates outside the laboratory [7]. These systems offer the potential of continuous, long-term monitoring, which is essential for patient follow-up, optimising sports performance, or controlling walking assistance exoskeletons [8,9,10].

Nevertheless, there are inherent challenges in designing accurate and cost-effective instrumented insoles, including the number, size and configuration of pressure sensors. Although high-resolution systems can provide detailed pressure maps, they generate a considerable volume of data, and are expensive to manufacture and complex to analyse, thus limiting their large-scale applicability [11]. These constraints have stimulated growing interest in optimising the number and placement of sensors to balance CoP measurement accuracy and system practicality [12,13].

Research has therefore focused on determining optimal sensor configurations. Regarding the number of sensors, previous studies highlight that optimal density is inherently task-dependent, as demonstrated by force data analysis [14]. Specifically for gait analysis, recent findings suggest that a relatively high number of sensors, such as 11 to 13, is required to maintain accurate CoP trajectories [13]. Conversely, overly reduced layouts can compromise reliability; for instance, a predefined 9-sensor configuration was shown to produce large CoP errors during balance tasks [6]. Nevertheless, for specific low-cost or Internet of Things (IoT) applications, some studies have explored highly reduced configurations ranging from 3 to 7 sensors [11,15], highlighting the constant trade-off between accuracy and hardware complexity. Similarly, various strategies have been proposed to determine the optimal spatial placement of these sensors. Early analytical methods utilized regressional approaches, such as stepwise inclusion, to identify the most relevant anatomical areas [16]. More recently, placements have been guided by heuristic anatomical foot models [12,17] or explored through artificial intelligence, utilizing genetic algorithms [18] and deep reinforcement learning [19]. Despite these advancements, the literature lacks a consensus on the exact spatial placement required for optimal CoP tracking. Most studies rely on heuristic placements based on anatomical assumptions or evaluate a very limited set of predefined configurations. Furthermore, the decoupling of stability axes (e.g., antero-posterior vs. medio-lateral) in sensor placement strategies remains under-investigated.

In this context, the main objective of this study is to simultaneously determine the optimal sensor number and placement for CoP estimation in instrumented insoles. Specifically, we aim to identify the most efficient subsets from an initial high-density matrix to minimize CoP computation errors along the antero-posterior, medio-lateral, and global Euclidean directions. Unlike classical approaches that focus on a single predefined layout, this study proposes an exhaustive evaluation of the trade-off between sensor count and estimation accuracy. By identifying the critical inflection point of performance and introducing a selection frequency analysis, this work provides data-driven, practical recommendations for the placement of sensors, ultimately guiding the design of energy-efficient and cost-effective wearable gait monitoring systems.

## 2. Materials and Methods

### 2.1. Participants

Data were collected from a cohort of 12 healthy volunteers (age: 38.67±16.08 years; height: 177±6 cm; weight: 72.32±9.67 kg; Body Mass Index (BMI): 23.17±3.32 kg/m^2^). Detailed anthropometric characteristics for each participant are provided in Appendix A (Table A1). This demographic data collection ensured a representative range of foot sizes and body mass indices, which are critical factors influencing plantar pressure distribution. None of the participants reported any history of musculoskeletal or neurological disorders affecting gait. All participants provided informed consent prior to the study, which was conducted in accordance with the Declaration of Helsinki.

### 2.2. Instrumentation

**Figure 1:**
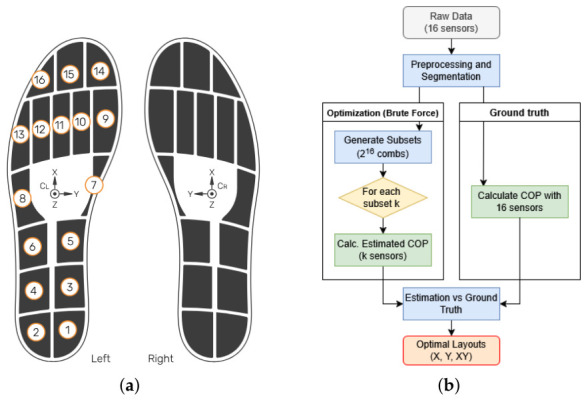
Experimental workflow and data processing pipeline. (**a**) Layout of the 16 capacitive pressure sensors on the smart insole (adapted from [20]). Note that sensor numbering is illustrated on the left insole only; the right insole employs a strictly mirrored numbering convention. (**b**) Exhaustive combinatorial search framework used to identify the optimal sensor subsets.

Gait data were recorded using connected pressure insoles (OpenGo, Moticon®, 100 Hz, 16 pressure cells covering 65 % of the insole area with a N·cm^−2^ resolution and a hysteresis < 1% [20]). Two insole sizes (40–41 and 42–43 EU sizes) were used to accommodate the participants’ foot anthropometry. The precise positions $\left(x_{i},y_{i}\right)$ and surface areas $A_{i}$ of each sensor *i* (Si) were known and fixed for each size, corresponding to the anatomical regions detailed in Table 1. The overall experimental workflow and sensor layout are illustrated in Figure 1.

### 2.3. Experimental Protocol

During the trials, participants were instructed to walk in a straight line on a flat, rigid surface. They were asked to walk at their comfortable, self-selected walking speed. This condition was chosen to capture the most natural gait patterns and minimize variability induced by imposed cadences. Recording durations were adjusted to ensure a sufficient number of gait cycles. To guarantee that only steady-state walking was analyzed, the continuous data streams were cropped by systematically discarding the first and last steps of each recording. This process effectively removed the initial acceleration and terminal deceleration phases, yielding a range of 27 to 49 continuous steps per subject (mean: 35.5±6.4 steps) that were included in the subsequent statistical analysis.

### 2.4. Data Preprocessing and Gait Segmentation

Raw pressure data were processed using a custom Python script. First, missing data points were handled using linear interpolation (limit: 5 frames). A zero-order hold was applied to pressure channels where no contact was detected.

**Figure 2:**
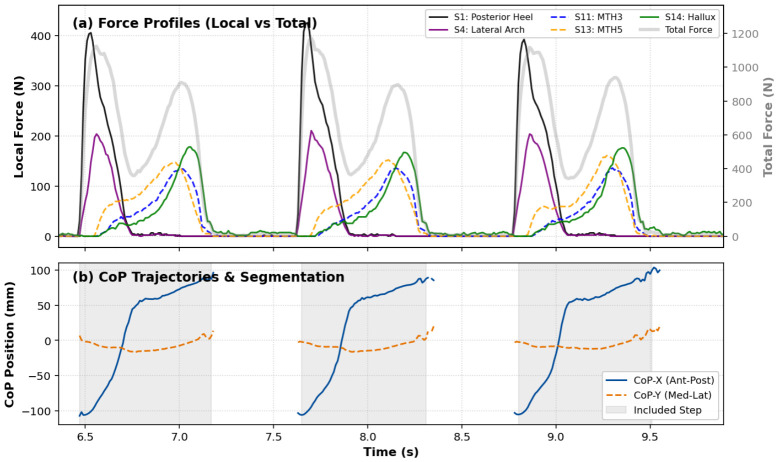
Signal Quality and Segmentation Validation. (**a**) Sensor Activation Sequence: Temporal evolution of vertical forces for five representative sensors. The sequential peaks (S1→S4→S13→S11→S14) illustrate a natural heel-to-toe roll-over, explicitly confirming the recruitment of the Lateral Arch (S4, purple) during the transition phase. The gray background curve represents the Total Force, validating the 15 N detection threshold. (**b**) CoP Trajectories: The Antero-Posterior ($CoP_{X}$, solid blue) and Medio-Lateral ($CoP_{Y}$, dashed orange) displacements are shown. Gray shaded areas indicate the gait cycles retained by the segmentation algorithm.

A biomechanical segmentation algorithm was developed to isolate individual steps from the continuous signal. A step was included in the analysis if it represented a complete, uninterrupted stance phase surrounded by swing phases (periods of zero pressure). As illustrated in Figure 2, a stance phase had to meet two concurrent criteria:

- Force Threshold: The total vertical ground reaction force must exceed 15 N for a continuous duration of at least 200 ms to exclude artifactual noise spikes.
- CoP Progression: A physiological heel-to-toe roll-over must be detected using a peak detection algorithm on the Antero-Posterior displacement ($CoP_{X}$). Specifically, the $CoP_{X}$ trajectory must initiate in the posterior third of the insole (rearfoot) and progress monotonically towards the anterior third (forefoot/toes) to ensure the participant completed a full physiological roll-over rather than a shuffling movement.

Figure 2a demonstrates the signal quality, showing the distinct sequential activation of the sensors from the heel (S1) through the lateral arch (S4) to the hallux (S14). This validates that the segmentation logic (gray shaded areas in Figure 2b) correctly captures dynamic gait cycles while excluding static periods or shuffling.

### 2.5. CoP Calculation (Ground Truth)

**Figure 3:**
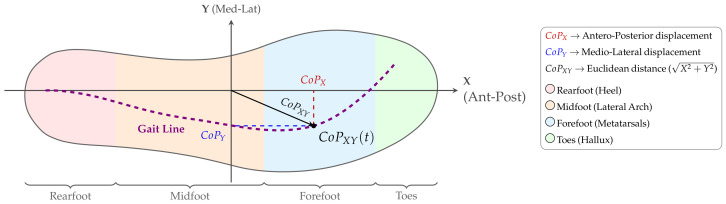
Coordinate System, Functional Segmentation, and CoP Metrics. The insole geometry is visualized in the dimensionless Coordinate System (COS). (1) Axes: The X-axis represents the Antero-Posterior displacement, while the Y-axis represents the Medio-Lateral displacement. (2) Anatomy: The foot is segmented into four functional zones: Rearfoot, Midfoot, Forefoot, and Toes. (3) Dynamics: The dashed violet line illustrates the CoP trajectory (Gait Line).

The anatomical zones and coordinate system used for these calculations are detailed in Figure 3. The CoP was calculated using the weighted barycenter method. For a given time *t*, the CoP coordinates $\left(CoP_{x},CoP_{y}\right)$ are defined as:

(1) $$CoP_{x}\left(t\right)=\frac{\sum_{i=1}^{N}P_{i}\left(t\right)\cdot A_{i}\cdot x_{i}}{\sum_{i=1}^{N}P_{i}\left(t\right)\cdot A_{i}}\;,\;CoP_{y}\left(t\right)=\frac{\sum_{i=1}^{N}P_{i}\left(t\right)\cdot A_{i}\cdot y_{i}}{\sum_{i=1}^{N}P_{i}\left(t\right)\cdot A_{i}}$$

where:

- N=16 is the total number of sensors.
- $P_{i}\left(t\right)$ is the pressure value of sensor *i* at time *t*.
- $A_{i}$ is the surface area of sensor Si.
- $\left(x_{i},y_{i}\right)$ are the geometric coordinates of the centroid of sensor Si.

This 16-sensor configuration served as the Ground Truth (GT) reference for evaluating the performance of reduced sensor subsets. This configuration was used as a reference rather than an absolute ground truth, given the absence of higher-resolution measurements in this setup.

### 2.6. Combinatorial Optimization Strategy

To identify the optimal sensor layout, we performed an exhaustive combinatorial search. We tested all possible combinations of *k* sensors among the 16 available, for *k* ranging from 1 to 15. The total number of combinations tested was $2^{16}-1=65,535$.

For each combination $C_{k}$ (a subset of *k* sensors), the CoP was recalculated ($CoP_{est}$) using only the pressure data from the selected sensors, while the weights of the excluded sensors were set to zero.

The optimization was performed separately for three distinct targets to assess whether different anatomical zones govern different stability axes:

1. Antero-Posterior accuracy (CoP-X): Minimizing the error on the X-axis.
2. Medio-Lateral accuracy (CoP-Y): Minimizing the error on the Y-axis.
3. Global accuracy (CoP-XY): Minimizing the 2D Euclidean distance.

### 2.7. Performance Metrics and Statistical Analysis

The accuracy of each combination was quantified using the Root Mean Square Error (RMSE) relative to the Ground Truth, calculated over all included gait frames across all subjects.

(2) $$RMSE=\sqrt{\frac{1}{T}\sum_{t=1}^{T}{\left(CoP_{GT}\left(t\right)-CoP_{est}\left(t\right)\right)}^{2}}$$

RMSE was computed independently for the antero-posterior ($CoP_{X}$) and medio-lateral ($CoP_{Y}$) axes, and as the Euclidean distance for the global ($CoP_{XY}$) metric.

To assess the robustness of the reduced layouts, we also computed the 95th percentile (P95) of the error distribution, representing the worst-case performance in realistic conditions.

Finally, a Marginal Gain Analysis was conducted to determine the optimal number of sensors. The inflection point of the performance curve was identified as the point where adding an extra sensor yielded a marginal accuracy gain of less than 1.5 mm.

All analyses were performed using Python 3.9 (libraries: pandas, numpy, scipy, matplotlib).

## 3. Results

### 3.1. Accuracy Convergence and Marginal Gain Analysis

**Figure 4:**
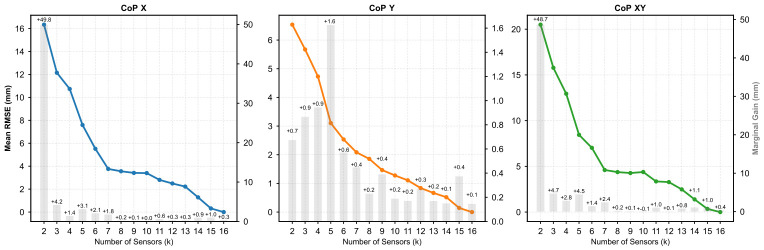
Performance trends for CoP-X (Antero-Posterior), CoP-Y (Medio-Lateral), and CoP-XY (Global) over the full optimization range (k=2 to 16). The solid lines represent the Mean RMSE (left axis), and the grey bars quantify the marginal accuracy gain obtained by adding an extra sensor (right axis).

The evolution of the CoP estimation accuracy as a function of the number of sensors (*k*) is illustrated in Figure 4. Mathematically, the largest absolute reductions in RMSE for the antero-posterior axis ($CoP_{X}$) and global Euclidean distance ($CoP_{XY}$) occur early in the optimization process (e.g., transitioning to k=3). However, the absolute errors at these minimal densities remain high (RMSE > 10 mm). A subsequent distinct reduction in the convergence curve is observed at the transition from k=4 to k=5. At this specific step, $CoP_{Y}$ experiences its maximum discrete error reduction (+1.6 mm), and the global $CoP_{XY}$ error drops to 8.45 mm. Beyond k=7, where the RMSE reaches a threshold below 5 mm for $CoP_{X}$ and $CoP_{XY}$, the marginal improvements fall below 1.2 mm per added sensor. Conversely, the $CoP_{Y}$ error does not plateau at k=7 but continues to show steady, albeit smaller, reductions up to k=15.

### 3.2. Anatomical Distribution of Optimal Sensors

**Figure 5:**
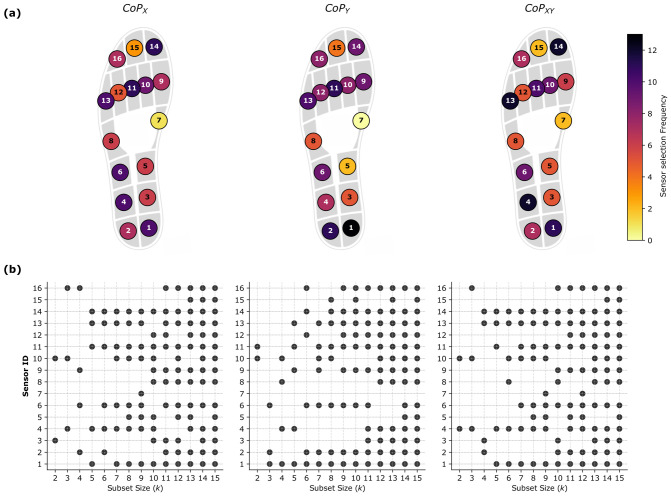
Anatomical Relevance and Subset Composition. (**a**) Selection Frequency Heatmap: Illustration of the recurrence of each sensor across the optimal subsets (k=2 to 15). The color intensity (from yellow to black) represents the number of times a sensor was selected, highlighting the dominance of the Heel (S1) and Lateral Arch (S4) zones for $CoP_{X}$ estimation. (**b**) Optimal Combination Composition: Detailed breakdown of the sensors selected for each subset size (*k*). The grid allows for identification of the specific sensors constituting the optimal solution for any given complexity level. The persistent selection of specific sensors confirms their structural importance.

Figure 5 illustrates the frequency of sensor selection within the optimal subsets across various subset sizes (*k*). For the longitudinal axis ($CoP_{X}$), the most frequently selected sensors across all subset sizes are the 3rd Metatarsal Head (S11) and the Hallux (S14), closely followed by the Posterior Medial Heel (S1), the Anterior Lateral Heel and Midfoot (S4, S6), and the 5th Metatarsal Head (S13). For the medio-lateral axis ($CoP_{Y}$), the Posterior Medial Heel (S1) is the most recurrent, being selected in all subsets for k≥3. It is followed by the Posterior Lateral Heel (S2) and the 3rd Metatarsal Head (S11), which also show high selection frequencies. For the global optimization ($CoP_{XY}$), the most frequently selected sensors are the Anterior Lateral Heel (S4), the Posterior Medial Heel (S1), the 5th Metatarsal Head (S13), and the Hallux (S14).

### 3.3. Spatial Robustness and Error Mapping

Table 2 and Table 3 detail the RMSE achieved by configurations optimized specifically for the Antero-Posterior (*X*), Medio-Lateral (*Y*), and Global (XY) targets, for k=5 and k=7 respectively.

As observed in both tables, the 5-sensor and 7-sensor configurations optimized for the global $CoP_{XY}$ target are identical to those optimized solely for the $CoP_{X}$ target. In terms of cross-performance for k=5, optimizing strictly for the medio-lateral axis (*Y*) reduces the $CoP_{Y}$ RMSE to 3.10 mm but increases the $CoP_{X}$ RMSE to 14.37 mm. Conversely, the configuration optimized for both *X* and XY yields a $CoP_{Y}$ RMSE of 3.70 mm and a $CoP_{X}$ RMSE of 7.60 mm. A similar trend is observed for k=7.

**Figure 6:**
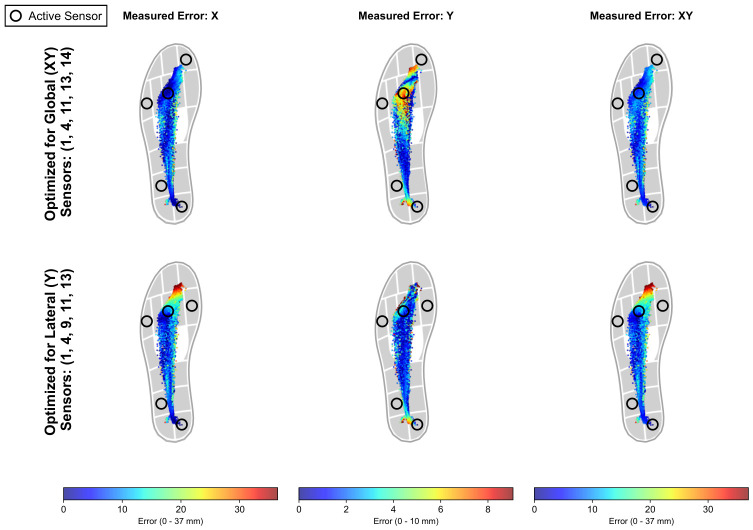
Cross-spatial error analysis for the 5-sensor configurations. The top row illustrates the local absolute error maps when using the layout optimized for the global $CoP_{XY}$ target (Sensors 1, 4, 11, 13, 14). The bottom row displays the error maps for the layout optimized strictly for the medio-lateral $CoP_{Y}$ target (Sensors 1, 4, 9, 11, 13). Columns separate the measured error on the *X*-axis, *Y*-axis, and the global XY Euclidean distance. Independent color scales, thresholded at the 99th percentile of the error distribution, are used for each column to accommodate the vastly different magnitudes between the antero-posterior and medio-lateral displacements. Active sensors for each layout are highlighted with black circles.

Figure 6 visually corroborates these results for the 5-sensor configurations. The figure compares the spatial error distribution (along the *X*-axis, *Y*-axis, and global XY Euclidean distance) for the two distinct layouts identified:

- Layout optimized for $CoP_{X}$ and $CoP_{XY}$ (Top Row): Using sensors 1, 4, 11, 13, and 14, the error magnitude remains predominantly low (<10 mm) across the central midstance area across all three error maps. Higher error values (>8 mm) are highly localized at the posterior heel edge and the distal forefoot/hallux region.
- Layout optimized for $CoP_{Y}$ (Bottom Row): Using sensors 1, 4, 9, 11, and 13, the *Y*-error map displays minimal errors throughout the stance phase. However, the *X*-error and XY-error maps reveal extensive regions of elevated error (>8 mm) at the longitudinal extremities, particularly across the entire anterior forefoot and toe regions. This spatial distribution reflects the increased $CoP_{X}$ RMSE observed in Table 2.

## 4. Discussion

By explicitly coupling the reduction of sensor number with an exhaustive analysis of sensor placement, this study addresses a gap in the literature. While previous works have successfully demonstrated that low-density arrays can achieve acceptable CoP accuracy, they often rely on predefined anatomical masks or evaluate a limited set of configurations. For instance, Chou et al. [11] evaluated reductions from 89 to 11 sensing points using predefined regional masks, concluding that 36 sensors were necessary for optimal accuracy while 11 sensors yielded the worst performance. Similarly, Fuchs et al. [13] simulated layouts ranging from 3 to 17 sensors and concluded that a minimum of 11 to 13 sensors is required to achieve high concordance (CCC ≥ 0.95) across different gaits. Our data-driven approach not only challenges these numerical thresholds but extends current knowledge by demonstrating that comparable or superior accuracy can be achieved with only 5 to 7 sensors by abandoning heuristic placements.

The primary objective of this study was to identify optimal sparse sensor layouts using an exhaustive combinatorial approach. Unlike heuristic methods that typically assume a linear relationship between sensor count and estimation accuracy, our results reveal a non-linear, multi-stage convergence pattern, corroborating the need for advanced optimization techniques [18,19].

### 4.1. Biomechanical Interpretation of Optimization Thresholds

A primary finding of this study is the identification of distinct, non-linear thresholds in estimation accuracy, revealing a critical trade-off between sensor density, clinical viability, and the specific directional axis being targeted (Figure 4).

To contextualize the performance of our models, recent literature reports varying CoP estimation errors using low-density arrays. Using an improved genetic algorithm to optimize an 8-sensor layout, Xian et al. [18] reported a mean absolute error of 3.81 mm in the medio-lateral direction ($CoP_{Y}$) and 8.61 mm in the antero-posterior direction ($CoP_{X}$). Furthermore, Fuchs et al. [13] evaluated configurations ranging from 3 to 17 sensors and identified a 13-sensor layout as the optimal compromise. For this specific 13-sensor configuration, they reported root mean square errors (RMSE) during walking of 6 mm for $CoP_{Y}$ and 19 mm for $CoP_{X}$.

In contrast to approaches that purely minimize absolute mathematical variance, our analysis identifies optimal thresholds based on biomechanical and clinical viability. As established in the results, while the largest absolute reductions in RMSE occur at minimal densities (k≤3), the residual errors remain prohibitive for clinical use (>10 mm). The primary inflection point in the optimization curve, which reduces the estimation error to a clinically applicable magnitude, is observed at the transition from four to five sensors. At this specific step, lateral stability ($CoP_{Y}$) experiences its maximum discrete error reduction, dropping to an RMSE of 3.1 mm. This is lower than the errors reported for the 13-sensor configuration in Fuchs et al. [13] for medio-lateral balance.

Biomechanically, minimal configurations (k≤4) typically cover only the extreme boundaries of the foot, failing to capture complex midfoot kinematics and effectively modeling the foot as a single rigid segment [1]. The inclusion of a fifth sensor, strategically located in the central metatarsal region (typically S11), acts as a central pivot. This captures the deformation of the transverse arch and the fine pronation and supination adjustments that occur during the midstance phase [15]. For k=5, the uniform accuracy across the central contact area confirms that reducing spatial discontinuities between the lateral loading column and the medial propulsion column is the most effective modification to secure reliable CoP tracking.

A secondary stabilization threshold is achieved at k=7, representing the definitive inflection point for longitudinal and global progression. At this density, the $CoP_{X}$ and $CoP_{XY}$ errors drop below the 5 mm clinical threshold (achieving 3.76 mm for $CoP_{X}$). This improvement is driven by sensor redundancy in the propulsion zones, reinforcing the lateral midfoot (S6) and densifying the metatarsal line (S10). This added density allows the estimation algorithm to mathematically decouple the medial column (push-off) from the lateral column (balance). Reaching this specific sensor density yields a reliable CoP trajectory suitable for standard clinical gait monitoring [8], while utilizing nearly half the sensors recommended by previous optimal layouts [13].

### 4.2. Anatomical Relevance and the Role of the Posterior Heel

The anatomical selection frequency analysis prompts a critical re-evaluation of conventional insole design methodologies, which frequently prioritize the posterior heel as the primary anchor for extracting all spatiotemporal gait parameters [7,12]. While our data-driven approach confirms the necessity of the heel, it reveals a distinct functional dichotomy depending on the targeted stability axis.

- Longitudinal Tracking ($CoP_{X}$): When optimizing for the antero-posterior axis, the algorithm systematically allocates the majority of active sensors to the anterior regions. While a posterior heel anchor (predominantly the medial heel, S1, occasionally shifting to the lateral heel, S2, as observed at k=6) is retained for subsets k≥5, the optimization relies heavily on the lateral arch (S4) and specific forefoot/toe regions—most notably the 3rd metatarsal head (S11), the 5th metatarsal head (S13), and the hallux (S14). This spatial distribution is consistent with the temporal distribution of load during stance. The initial heel strike, while essential for impact detection, constitutes a relatively brief transient phase (typically 10-15% of the stance duration) [1]. In contrast, the CoP resides significantly longer in the midfoot and forefoot regions during the sustained midstance and propulsion phases. To minimize the cumulative spatial error over the entire gait cycle, the optimization algorithm inherently densifies the sensor network along this prolonged forward progression path.
- Lateral Stability ($CoP_{Y}$): Conversely, the rearfoot region proved consistently required for medio-lateral accuracy. The posterior medial heel (S1) is selected almost universally across subsets (k≥3), frequently accompanied by the posterior lateral heel (S2). This indicates that capturing the initial lateral balance anchor upon foot strike dictates the subsequent accuracy of the entire medio-lateral trajectory.

Ultimately, this functional decoupling demonstrates that plantar pressure measurement systems should consider stability axes independently: capturing continuous forward progression (*X*-axis) requires a robust metatarsal and arch distribution, whereas tracking lateral balance (*Y*-axis) mandates a highly densified posterior heel cluster.

### 4.3. Application-Specific Layouts and Directional Error Trade-Offs

The cross-performance analysis (Table 2 and Table 3) highlights a fundamental consideration for hardware design: the necessity for application-specific sensor selection. As demonstrated, the global estimation error ($CoP_{XY}$) is heavily dominated by the antero-posterior axis ($CoP_{X}$). This is a direct consequence of the elongated geometry of the human foot and the large amplitude of forward progression during gait compared to lateral sway [11].

In practical applications, this geometric dominance implies that a general XY-optimized layout is effective for standard gait analysis tracking forward progression. However, this configuration is less effective for clinical assessments focusing primarily on lateral stability, such as fall risk evaluation in elderly populations or post-stroke balance monitoring [1,8]. In such contexts, shifting to a strictly *Y*-optimized layout provides improved fidelity in capturing critical medio-lateral sway, albeit by significantly compromising antero-posterior accuracy. For instance, at a 7-sensor density, prioritizing the *Y*-axis reduces the medio-lateral error to its absolute minimum but induces a severe degradation of the longitudinal tracking, increasing the $CoP_{X}$ RMSE from 3.76 mm to over 14.30 mm. This quantified trade-off highlights that a single generalized layout is unlikely to optimally serve all diagnostic purposes.

### 4.4. Practical Implications for Wearable Sensor Design

These findings provide a modular framework for hardware design based on the primary clinical axis of interest. Rather than a single optimal density, designers must tailor the sensor array to the specific diagnostic target:

1. Basic Temporospatial Tracking (k=5, XY-Optimized): A general configuration focusing on the posterior heel, lateral arch, third and fifth metatarsal heads, and hallux (S1,S4,S11,S13,S14) provides a robust overall trajectory estimation (global RMSE of 8.45 mm). This configuration represents the first functionally viable threshold, sufficient for basic temporospatial parameter extraction but lacking the precision required for rigorous clinical asymmetry diagnostics.
2. Advanced Longitudinal Gait Assessment (k=7, XY-Optimized): To achieve high-fidelity tracking of forward progression, upgrading the array to 7 sensors by integrating the central/lateral midfoot (S6) and densifying the metatarsal line (S10) is the most efficient modification. This reduces the global RMSE to 4.60 mm and the $CoP_{X}$ RMSE to 3.76 mm. As this density represents an inflection point for the longitudinal axis, adding further sensors yields marginal gains relative to the increased hardware complexity.
3. Lateral Stability and Balance Diagnostics (*Y*-Optimized): If the primary clinical focus is medio-lateral sway (e.g., postural control or fall risk), the design architecture must be fundamentally altered. A 5-sensor, strictly *Y*-optimized layout focusing on the heel and metatarsal region (S1,S4,S9,S11,S13) already achieves a highly precise RMSE of 3.10 mm. Furthermore, unlike the longitudinal axis, the medio-lateral convergence curve lacks a strict inflection point at higher densities, showing steady, continuous improvements up to 15 sensors. Consequently, for lateral diagnostics, designers should determine the sensor count based strictly on the required clinical tolerance rather than a mathematical stabilization plateau.

### 4.5. Limitations

It is important to acknowledge that this optimization process minimizes the average spatial error (RMSE) over the entire stance phase. Consequently, it naturally prioritizes longer gait phases over rapid transient events. For applications specifically targeting impact transient analysis, utilizing a weighted cost function that emphasizes the first 50 ms of the gait cycle might yield different optimal layouts. Additionally, as these results are derived from healthy gait patterns, pathological foot deformities (e.g., pes planus) may alter the anatomical relevance of the arch sensor [15].

Furthermore, this study investigated walking exclusively at a self-selected, comfortable speed. Previous research has demonstrated that CoP estimation accuracy in simplified pressure insoles is highly task-dependent and varies significantly across different walking speeds [13]. Future work should validate these optimal placements under varying dynamic conditions, such as fast walking, running, or load carriage, to ensure the robustness of the sensor layouts across a broader range of functional tasks.

## 5. Conclusions

This study demonstrates that the hardware design of instrumented insoles can be significantly streamlined without severely compromising CoP tracking accuracy, provided that sensor placement is meticulously tailored to the specific biomechanical axis of interest. Our exhaustive combinatorial analysis reveals that targeted spatial distribution, rather than absolute sensor density, is the primary driver of estimation fidelity. A central conclusion of this work is the functional decoupling of stability axes. We established that longitudinal tracking ($CoP_{X}$) relies heavily on midfoot transitions and metatarsal boundaries to capture continuous forward progression, whereas medio-lateral balance ($CoP_{Y}$) is strictly anchored to the posterior heel region. Consequently, universal heuristic layouts are inherently sub-optimal, and hardware design must be intrinsically linked to the primary clinical diagnostic target. Based on our quantitative findings and the observed convergence thresholds, we conclude that a minimum of 7 strategically placed sensors is recommended to achieve robust, clinically viable CoP tracking across all spatial axes. While our data confirms that increasing the sensor count beyond k=7 continues to yield steady mathematical improvements—particularly for medio-lateral sway ($CoP_{Y}$)—the 7-sensor configuration represents a clear inflection point where sub-centimetric accuracy is secured. Moving beyond this baseline introduces diminishing marginal returns in CoP estimation accuracy relative to the substantially increased hardware and computational complexity. Ultimately, this study provides a quantitative, data-driven framework that prioritizes strategic sensor placement, guiding the development of the next generation of cost-effective, task-specific, and energy-efficient wearable gait analysis systems. 

## Figures and Tables

**Table 1:** Translation table mapping sensor numbers to their corresponding anatomical regions and functional zones.

Sensor Number	Anatomical Region
Zone 1: Rearfoot (Initial contact and lateral stability)	
1	Posterior Medial Heel
2	Posterior Lateral Heel
3	Anterior Medial Heel
4	Anterior Lateral Heel/Lateral Arch
Zone 2: Midfoot (Transition phase)	
5	Central/Medial Midfoot
6	Central/Lateral Midfoot
7	Medial Arch
8	Lateral Midfoot Border
Zone 3: Forefoot (Propulsion phase)	
9	1st Metatarsal Head (MTH1)
10	2nd Metatarsal Head (MTH2)
11	3rd Metatarsal Head (MTH3)
12	4th Metatarsal Head (MTH4)
13	5th Metatarsal Head (MTH5)
Zone 4: Toes (End of propulsion)	
14	Hallux (Great Toe)
15	Central Toes
16	Lateral Toes

**Table 2:** Cross-performance of the optimal 5-sensor configurations. Values in bold represent the minimum error for each target.

Optimized Target	Selected Sensors	RMSE ${\mathrm{CoP}}_{X}$ (mm)	RMSE ${\mathrm{CoP}}_{Y}$ (mm)	RMSE ${\mathrm{CoP}}_{\mathrm{XY}}$ (mm)
Antero-Posterior (*X*)	1, 4, 11, 13, 14	**7.60**	3.70	**8.45**
Medio-Lateral (*Y*)	1, 4, 9, 11, 13	14.37	**3.10**	14.70
Global (XY)	1, 4, 11, 13, 14	**7.60**	3.70	**8.45**

**Table 3:** Cross-performance of the optimal 7-sensor configurations. Values in bold represent the minimum error for each target.

Optimized Target	Selected Sensors	RMSE ${\mathrm{CoP}}_{X}$ (mm)	RMSE ${\mathrm{CoP}}_{Y}$ (mm)	RMSE ${\mathrm{CoP}}_{\mathrm{XY}}$ (mm)
Antero-Posterior (*X*)	1, 4, 6, 10, 11, 13, 14	**3.76**	2.65	**4.60**
Medio-Lateral (*Y*)	1, 2, 6, 9, 10, 11, 13	14.30	**2.09**	14.45
Global (XY)	1, 4, 6, 10, 11, 13, 14	**3.76**	2.65	**4.60**

## Data Availability

Dataset available on request from the authors.

## Abbreviations

The following abbreviations are used in this manuscript:

CoP	Center of Pressure
RMSE	Root Mean Square Error
GT	Ground Truth
MTH	Metatarsal Head (e.g., MTH1, MTH5)
BMI	Body Mass Index

## Appendix A. Participant Characteristics

The detailed anthropometric data for the volunteers involved in the study are listed below.

**Table A1:** Detailed anthropometric characteristics of the participants.

Subject ID	Gender	Age (Year)	Height (m)	Weight (kg)	BMI (kg/m^2^)
S01	F	54	1.69	66.0	23.1
S02	M	56	1.80	65.0	20.1
S03	M	32	1.80	66.0	20.4
S04	F	47	1.74	91.0	30.1
S05	M	29	1.78	83.2	26.3
S06	M	20	1.70	62.6	21.7
S07	M	50	1.81	81.0	24.7
S08	F	29	1.74	77.0	25.4
S09	M	69	1.78	73.0	23.0
S10	M	24	1.71	73.0	25.0
S11	M	31	1.77	57.0	18.2
S12	M	23	1.90	73.0	20.2
Mean	-	38.67	1.77	72.32	23.17
SD	-	16.08	0.06	9.67	3.32

## Appendix B. Detailed Optimization Statistics

**Table A2:** Detailed CoP-X Statistics.

Num	Combo	RMSE	Std	Min	Max	P95	CI Low	CI High
1	7	66.18	24.35	0.01	116.92	93.90	54.12	55.24
2	3, 10	16.31	9.02	0.00	60.00	29.83	14.97	15.27
3	4, 10, 16	12.02	7.67	0.00	54.04	24.71	11.89	12.14
4	2, 6, 9, 16	10.70	6.03	0.00	48.56	19.53	10.60	10.80
5	1, 4, 11, 13, 14	7.60	4.58	0.00	34.82	14.73	7.52	7.67
6	2, 4, 6, 11, 13, 14	5.44	3.54	0.00	34.09	11.04	5.38	5.50
7	1, 4, 6, 10, 11, 13, 14	3.76	2.52	0.00	31.97	7.49	3.72	3.81
8	1, 4, 5, 6, 10, 11, 13, 14	3.56	2.25	0.00	31.97	7.06	3.52	3.60
9	1, 4, 5, 6, 7, 10, 11, 13, 14	3.42	2.18	0.00	31.97	6.80	3.38	3.45
10	1, 3, 4, 5, 8, 9, 10, 11, 12, 14	3.40	2.11	0.00	31.97	6.39	3.37	3.44
11	1, 2, 3, 6, 8, 9, 11, 12, 13, 14, 16	2.81	1.71	0.00	12.28	5.23	2.78	2.83
12	1, 2, 3, 5, 6, 8, 9, 10, 11, 13, 14, 16	2.50	1.54	0.00	12.75	4.83	2.47	2.52
13	1, 2, 4, 5, 6, 8, 9, 11, 12, 13, 14, 15, 16	2.22	1.29	0.00	6.83	4.24	2.20	2.25
14	1, 2, 3, 4, 6, 8, 9, 10, 11, 12, 13, 14, 15, 16	1.29	1.04	0.00	7.72	3.00	1.27	1.30
15	1, 2, 3, 4, 5, 6, 8, 9, 10, 11, 12, 13, 14, 15, 16	0.32	0.29	0.00	3.51	0.59	0.31	0.32
16	1, 2, 3, 4, 5, 6, 7, 8, 9, 10, 11, 12, 13, 14, 15, 16	0.00	0.00	0.00	0.00	0.00	-	-

**Table A3:** Detailed CoP-Y Statistics.

Num	Combo	RMSE	Std	Min	Max	P95	CI Low	CI High
1	15	6.99	4.12	0.00	21.59	13.45	6.91	7.07
2	10, 11	6.69	3.84	0.00	27.23	12.75	6.62	6.75
3	1, 2, 6	5.80	4.09	0.00	31.53	12.55	5.72	5.87
4	1, 4, 8, 10	4.73	3.04	0.00	17.65	9.75	4.68	4.78
5	1, 4, 9, 11, 13	3.10	1.98	0.00	26.58	6.11	3.07	3.14
6	1, 2, 6, 12, 14, 16	2.53	1.56	0.00	13.50	5.06	2.51	2.56
7	1, 2, 6, 9, 10, 11, 13	2.09	1.31	0.00	20.82	3.78	2.06	2.11
8	1, 2, 6, 10, 11, 13, 14, 15	1.86	1.10	0.00	10.92	3.53	1.84	1.87
9	1, 2, 6, 9, 11, 12, 13, 14, 16	1.47	0.88	0.00	6.11	2.78	1.45	1.48
10	1, 2, 6, 9, 11, 12, 13, 14, 15, 16	1.28	0.77	0.00	6.07	2.43	1.27	1.29
11	1, 2, 3, 4, 6, 9, 11, 12, 13, 14, 16	1.11	0.78	0.00	4.64	2.39	1.10	1.12
12	1, 2, 3, 4, 8, 9, 10, 11, 12, 13, 14, 16	0.84	0.54	0.00	4.02	1.59	0.83	0.85
13	1, 2, 3, 4, 8, 9, 10, 11, 12, 13, 14, 15, 16	0.67	0.50	0.00	2.37	1.43	0.66	0.68
14	1, 2, 3, 4, 5, 6, 8, 9, 10, 11, 12, 13, 14, 16	0.52	0.43	0.00	4.02	1.16	0.51	0.53
15	1, 2, 3, 4, 5, 6, 8, 9, 10, 11, 12, 13, 14, 15, 16	0.15	0.12	0.00	1.07	0.33	0.14	0.15
16	1, 2, 3, 4, 5, 6, 7, 8, 9, 10, 11, 12, 13, 14, 15, 16	0.00	0.00	0.00	0.00	0.00	-	-

**Table A4:** Detailed CoP-XY Statistics.

Num	Combo	RMSE	Std	Min	Max	P95	CI Low	CI High
1	5	57.63	27.08	7.04	120.51	98.12	57.01	58.24
2	4, 10	20.32	7.28	0.48	70.64	32.41	20.20	20.44
3	4, 10, 16	15.68	8.11	0.11	54.15	29.14	15.54	15.81
4	2, 3, 13, 14	12.91	5.88	0.09	40.66	21.28	12.81	13.01
5	1, 4, 11, 13, 14	8.45	4.04	0.00	36.35	15.00	8.38	8.52
6	1, 4, 8, 10, 13, 14	7.03	3.44	0.00	32.35	11.95	6.97	7.08
7	1, 4, 6, 10, 11, 13, 14	4.60	2.48	0.00	32.35	8.18	4.56	4.64
8	1, 4, 5, 6, 10, 11, 13, 14	4.37	2.26	0.00	32.35	7.80	4.34	4.41
9	1, 4, 5, 6, 7, 10, 11, 13, 14	4.27	2.22	0.00	32.35	7.61	4.23	4.31
10	1, 2, 3, 6, 8, 9, 11, 13, 14, 16	4.38	1.80	0.00	13.31	6.99	4.35	4.41
11	1, 2, 4, 5, 6, 9, 11, 12, 13, 14, 16	3.36	1.63	0.00	11.49	5.86	3.33	3.38
12	1, 2, 4, 5, 6, 7, 9, 11, 12, 13, 14, 16	3.28	1.60	0.00	11.49	5.77	3.25	3.31
13	1, 2, 3, 4, 6, 8, 9, 10, 11, 12, 13, 14, 16	2.48	1.45	0.00	8.93	4.49	2.46	2.51
14	1, 2, 3, 4, 6, 8, 9, 10, 11, 12, 13, 14, 15, 16	1.39	1.10	0.00	7.87	3.19	1.37	1.41
15	1, 2, 3, 4, 5, 6, 8, 9, 10, 11, 12, 13, 14, 15, 16	0.35	0.31	0.00	3.65	0.67	0.35	0.36
16	1, 2, 3, 4, 5, 6, 7, 8, 9, 10, 11, 12, 13, 14, 15, 16	0.00	0.00	0.00	0.00	0.00	-	-
